# 

**Published:** 1881-06

**Authors:** 


### CONTENTS.

| | Page. |
|---|---|
| Horseback Riding | 201 |
| God's Decree (poetry) | 203 |
| Nursing the Sick | 204 |
| From an Insect's Point of View | 207 |
| Short Talk on Protection | 208 |
| Decency Toward Horses | 209 |
| Dead in the Saddle | 210 |
| Removal of Stains and Spots | 211 |
| To Cure a Cold | 213 |
| Dieting for Health | 214 |
| Bad Temper and Insanity | 215 |
| When Began We ? | 218 |
| The Earliest Sign of Consumption | 222 |
| Dollars and Ideas | 224 |
| Cold Feet | 226 |
| Spring Diseases | 228 |
| The Earth's Great Age | 229 |
| Who Need Eye-Glasses | 230 |
| The Catacombs of Paris | 230 |
| Straw Lumber—A New Industry | 232 |
| Riding on the Rails | 233 |
| The Secret of Good Manners | 235 |
| A Cure for Car Sickness | 236 |

